# Jack-and-Master Trait Responses to Elevated CO_2_ and N: A Comparison of Native and Introduced *Phragmites australis*

**DOI:** 10.1371/journal.pone.0042794

**Published:** 2012-10-31

**Authors:** Thomas J. Mozdzer, J. Patrick Megonigal

**Affiliations:** Smithsonian Environmental Research Center, Edgewater, Maryland, United States of America; University of Georgia, United States of America

## Abstract

Global change is predicted to promote plant invasions world-wide, reducing biodiversity and ecosystem function. Phenotypic plasticity may influence the ability of introduced plant species to invade and dominate extant communities. However, interpreting differences in plasticity can be confounded by phylogenetic differences in morphology and physiology. Here we present a novel case investigating the role of fitness trait values and phenotypic plasticity to global change factors between conspecific lineages of *Phragmites australis*. We hypothesized that due to observed differences in the competitive success of North American-native and Eurasian-introduced *P. australis* genotypes, Eurasian-introduced *P. australis* would exhibit greater fitness in response to global change factors. Plasticity and plant performance to ambient and predicted levels of carbon dioxide and nitrogen pollution were investigated to understand how invasion pressure may change in North America under a realistic global change scenario. We found that the introduced Eurasian genotype expressed greater mean trait values in nearly every ecophysiological trait measured – aboveground and belowground – to elevated CO_2_ and nitrogen, outperforming the native North American conspecific by a factor of two to three under every global change scenario. This response is consistent with “jack and master” phenotypic plasticity. We suggest that differences in plant nitrogen productivity, specific leaf area, belowground biomass allocation, and inherently higher relative growth rate are the plant traits that may enhance invasion of Eurasian *Phragmites* in North America. Given the high degree of genotypic variability within this species, and our limited number of genotypes, our results must be interpreted cautiously. Our study is the first to demonstrate the potential importance of jack-and-master phenotypic plasticity in plant invasions when facing imminent global change conditions. We suggest that jack-and-master invasive genotypes and/or species similar to introduced *P. australis* will have an increased ecological fitness, facilitating their invasion in both stressful and resource rich environments.

## Introduction

Anthropogenic nitrogen (N) eutrophication and atmospheric CO_2_ enrichment are major anthropogenic global changes that affect plant invasion success [1]–[4]. Global changes such as these can alter the outcome of competition between native and introduced plants, potentially increasing the relative success of the introduced species [5]. Because C (as CO_2_) and N are often growth-limiting plant nutrients, responses to these factors will depend upon the traits that govern resource acquisition, the plasticity of these traits, and the genetic potential to acquire new traits via outcrossing. The presence or absence of specific plant traits that respond to global change factors will ultimately determine ecosystem responses to global change [6], but the link between plant invasion success and trait responses to global change perturbations has received little attention [7].

Phenotypic plasticity has been defined as the ability of a genotype to express different phenotypes under different environmental conditions [8] and can be a mechanism that facilitates plant invasions [9]. Although the role of phenotypic plasticity in determining plant responses to climate change is not well understood [7], a greater understanding of phenotypic plasticity may aid in predicting future invasions [10]. For example, phenotypic plasticity may either provide a buffer against rapid climate change [11] or enhance rapid adaptation of genotypes [12]. The identification of plants bearing highly plastic traits may allow *a priori* identification of plant species that are likely to be highly invasive [11].

Richards et al. [9] suggested that invasive plant species respond to differences in resource availability through three basic strategies. In comparison to an appropriate reference species, an invasive species that uses the ‘jack-of-all-trades’ strategy is more successful under unfavorable environmental conditions; a ‘master-of-some’ succeeds only under favorable environmental conditions; and a ‘jack-and-master’ succeeds under all conditions through a combination of a non-plastic fitness response under stressful conditions as well as a plastic fitness response under favorable conditions. Differences among plant species in their ability to respond to multiple global change variables are partly a consequence of differences in phenotypic and physiological plasticity.

Traditionally, comparisons of invasive and native species have been used to identify traits that promote the success of plant invasions and to forecast future invasions [5], [9], [13]. Such studies have often relied on congeneric comparisons. A disadvantage of such comparisons is that congeneric species differ phylogenetically, confounding ecological differences between genera in traits associated with invasiveness [14]. Although phylogenetic corrections can be made [15], the most robust and conservative approach is to assess phenotypic plasticity through the use of conspecific genetic lineages. In the present study, we compared genotypes of two conspecific lineages of the common reed *Phragmites australis* (hereafter *Phragmites*) in order to identify phenotypic and ecophysiological plant traits that respond to changes in resource availability, while minimizing the confounding influence of phylogeny on traits. By manipulating resource availability, we examined how genetically distinct genotypes differ in traits related to resource strategy, and attempted to predict how these traits will respond to future global change. Such an approach must be interpreted with some caution given the potential for intra-lineage differences.

*Phragmites* is cosmopolitan in distribution and is considered to be among the world's most widely distributed angiosperms [16]. The species includes over two dozen genetically distinct haplotypes world-wide [17]. The lineage native to the United States, *Phragmites australis americanus* (hereafter, native *Phragmites*) [18] has been present in North America for thousands of years [19], [20]. However, over the last fifty years, North American wetlands have been rapidly converted to near monocultures of a non-native Eurasian lineage *Phragmites australis australis* (hereafter, introduced *Phragmites*) [18], which was likely introduced in the 1800's [17]. The native and introduced lineages differ both morphologically [18] and physiologically [21]. When compared to the Atlantic coast native lineages, previous studies have found that the introduced lineage sustains greater photosynthetic rates [21], higher relative growth rates (RGR) [22], and greater N uptake rates [23]. To sustain such high growth, the introduced type requires nearly four times more N than the native type at extant densities [21]. It has been suggested that Atlantic coast natives are low-nutrient specialists due to higher N affinity at low concentrations and lower N demand [23], making the native type more competitive in low-nutrient environments [21].

As a C_3_ plant, *Phragmites* productivity should be stimulated by ongoing increases in the concentration of atmospheric CO_2_ [24]. However, the potential for increased growth at elevated CO_2_ can be limited by N availability [25]. In fact, exposure of a tidal marsh to elevated CO_2_ has been shown to dramatically reduce plant N availability [26], creating strong interactions between these key global change factors. Because the N economy of native and introduced *Phragmites* lineages are dramatically different [21], we hypothesized that these lineages may respond differently to increases in the availability of CO_2_ and N.

Here, we present an experimental test of the effects of elevated CO_2_ concentration and N eutrophication on native and introduced genetic lineages of the common reed, *Phragmites*. We sought to gain insights into the plasticity of plant traits that govern the growth, and presumably the invasion success, of the introduced *Phragmites* lineage under factorial scenarios of elevated CO_2_ and N eutrophication in a greenhouse study. This is the first study to our knowledge to assess trait plasticity in response to multiple interacting global change variables. We hypothesized that introduced *Phragmites* has a jack-and-master invasive strategy, responding more positively to elevated CO_2_ and N treatments –individually and interactively – than the native lineage and potentially other native species.

## Materials and Methods

The Atlantic coast native (*P. a. americanus* haplotype F) and introduced (*P. a. australis* haplotype M) *Phragmites* lineages used for the experiment were grown in a common garden at the University of Rhode Island (started in 2006) under identical conditions [27] and came from one genotype per lineage. These genotypes originated from two populations within 50 km of each other on the Delmarva Peninsula, USA; the native population originated from the upper St. Jones River in Dover, DE and the introduced originated from the Choptank River in Talbot County, MD. No specific permits were required for the described studies. We specifically tested for differences between genetic lineages, which due to our design equates to genotype. Given that geographic origin (population) was not found to have significant effects in a previous study comparing native and introduced *Phragmites* seedling response to N [28], we chose to replicate genotype to increase the precision of our trait estimates in response to global change. Because the genotypes were grown in a common environment for three years prior to the experiment, we assume that any differences observed between genotypes may be attributed to genetic differences alone. For each replicate, an individual rhizome fragment, containing 3–5 internodes (mean±SE = 1.29±0.07 g and 1.10±0.7 g dry weight for native and introduced, respectively) was planted on 11–12 June 2009 in 15 liter buckets (24 cm×24 cm×33 cm) (n = 96) filled to approximately 6 cm below the sediment surface with reed-sedge peat (Baccto, Houston, TX). There were no significant differences in starting rhizome mass between genotypes (p>0.05). After planting, four macro-pores were created by inserting a 1.25-cm *i.d.* PVC corer into the sediment to allow for water movement throughout the pot. Plants were kept inside a greenhouse at the Smithsonian Environmental Research Center until the first plant emerged on 19 June, after which individual plants were randomly assigned to a CO_2_ and N treatment, and transferred to one of six CO_2_ controlled chambers. After plant emergence, soils were kept saturated by adding tap water as needed to maintain at least 3 cm of inundation, simulating an anaerobic wetland environment. Final emergence from planted rhizome fragments was 55%, resulting in 52 replicate units (n = 27 native, n = 25 introduced).

Plants were exposed to one of four treatment combinations of CO_2_ and N. Chamber CO_2_ concentrations were maintained at either ambient or elevated CO_2_ (ambient CO_2_+330±23 ppmv). The chambers were similar to those used by Wolf *et al.* [29], but modified in height to have dimensions of 1.5 m length×0.9 m width×2.4 m height. Our experimental design was factorial with two levels of plant type (lineage), two levels of CO_2_ (ambient and elevated), and two levels of N (ambient and eutrophied). N-treated plants were given 3.1 g of NH_4_Cl diluted in 14 ml of deionized water approximately every two weeks throughout the experiment, resulting in a final application rate equivalent to 25 g N m^−2^ yr^−1^. This level of N loading is representative of eutrophied tidal marsh ecosystems [30]. Control plants were given 14 ml of deionized water in the same manner. Mean growing days from emergence to harvest was 58±1 d (mean ± SE). On July 21, plants in the elevated CO_2_+N treatment exhibited macronutrient deficiency and all mesocosms received 0.29 g of Potash (Espoma Quick Solutions) and 0.724 g Triple Phosphate (Espoma Quick Solutions), which resulted in a N-P-K (10∶1∶1) and (0∶1∶1) in the fertilization treatment and control treatment respectively. Tidal marshes where introduced *Phragmites* invade are generally considered to be N-limited ecosystems [31], and P or K deficiencies are not known to limit *Phragmites* invasion in North America.

Destructive harvest began on 20 August and continued through 27 August. A fully expanded leaf, third node from the top, was measured for leaf area using a LI-3000 leaf area meter (Li-Cor, Lincoln, NE, USA), and dry mass was recorded after freeze drying to determine the specific leaf area (SLA). The remaining leaves were then measured to determine total leaf area. Plant biomass was separated into one of four categories (leaves, stems, roots, and rhizomes) and oven dried at 60°C to constant mass. Leaf sheaths were calculated as part of the stem mass. Total plant production was calculated by subtracting starting rhizome mass (estimated from rhizome wet∶dry mass of unplanted samples) from total biomass. Plant tissue was homogenized and analyzed for total carbon (C) and N content using an elemental analyzer (CE Instruments NA2500, Milan, Italy). To evaluate growth parameters, biomass allocation, and the response of these variables to CO_2_ and N, we calculated relative growth rate (RGR), nitrogen productivity (NP), and leaf area ratio (LAR), using the growth equations in Lambers et al. [32]. To assess differences in biomass allocation, we calculated root mass fraction (RMF), rhizome mass fraction (RhMF), leaf mass fraction (LMF), and stem mass fraction (SMF) as the fraction of total biomass allocated to each category of plant material. Below∶above ground ratio was calculated as the ratio of belowground to aboveground biomass. Stimulation effects of treatments were calculated by subtracting the control responses from the manipulative treatment response, where treatment was elevated CO_2_ and/or high N, and control was the ambient CO_2_+low N treatment for each lineage.

To evaluate the effects of CO_2_ and N on plant lineage, we applied a 2×2×2 factorial analysis of variance using PROC GLM in SAS (SAS 9.1, Cary, NC). In a multi-level model where variance components for across- and within-chambers were estimated, across chamber variance was effectively 0. Therefore, we were able to combine information across the chambers (total pooling) [33]. Data were log-transformed when necessary to meet homogeneity of variance assumptions of ANOVA. We hypothesized *a priori* that introduced *Phragmites* would respond positively to both CO_2_ and N, and out-perform native *Phragmites* in terms of growth and the traits that favor high growth rates. We interpret significant lineage (G) interactions (G×CO_2_ and G×N) as lineage-specific (native vs. introduced) differences in trait plasticity to the response variable [9]. To evaluate how plant traits were affected by our experimental treatments, and how the different lineages were separated in trait space, a principal component analysis (PCA) was performed in SAS with PROC PRINCOMP using a correlation matrix of standardized data for the response variables.

## Results

**Figure 1:**
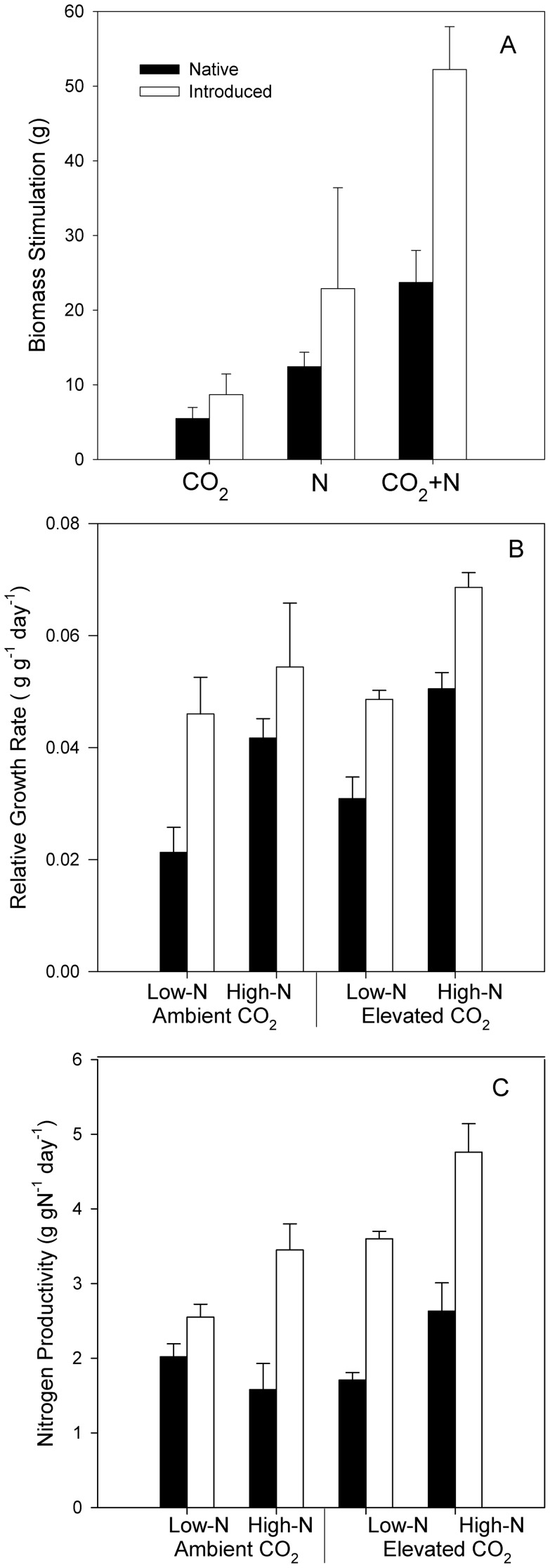
Effects of CO_2_ and N treatments on *Phragmites* biomass stimulation ± standard error (A), relative growth rate (RGR) ± standard error (B), and plant nitrogen productivity (NP) ± standard error (C). Solid bars indicate native *Phragmites* and empty bars indicated introduced *Phragmites*.

**Figure 2:**
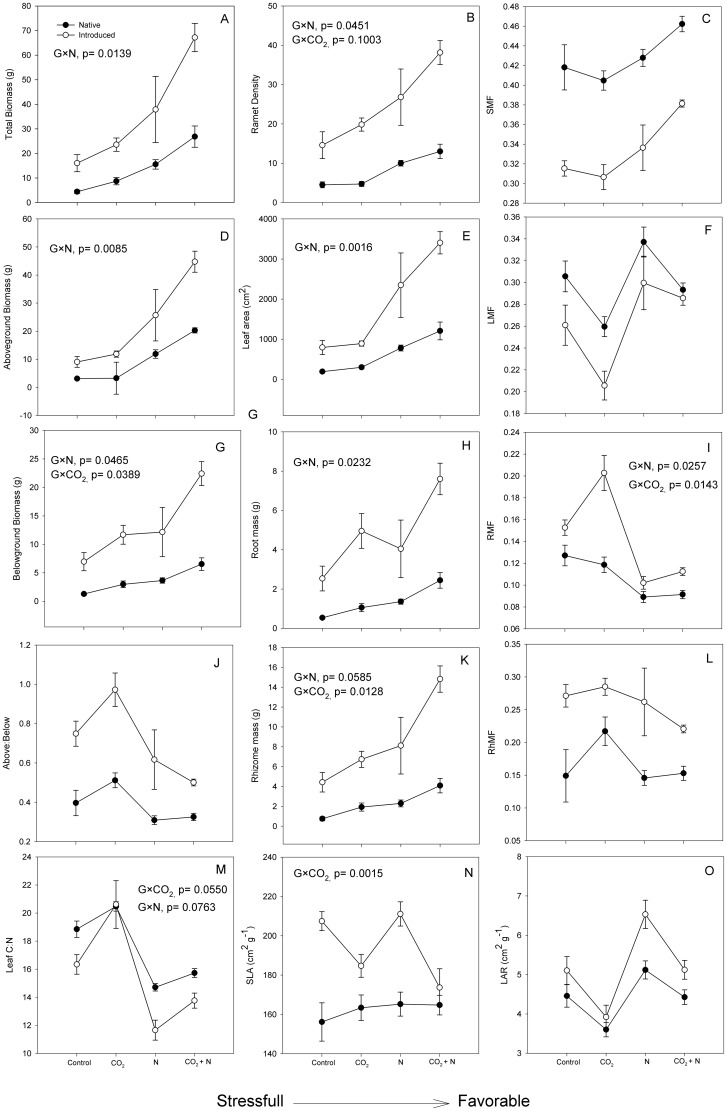
Interactive effects of CO_2_ and N on mean plant phenotype in native and introduced *Phragmites* demonstrating the phenotypic plasticity of the introduced type as the “Jack and Master” invader in A: total biomass, B: ramet density, C: stem mass fraction (SMF), D: aboveground biomass, E: leaf area, F: leaf mass fraction (LMF), G: belowground biomass, H: root mass, I: root mass fraction (RMF), J: belowground∶aboveground (BG∶AG), K: rhizome mass, L: rhizome mass fraction (RMF), M leaf C∶N, N: specific leaf area (SLA), O, leaf area ration (LAR). Significant G×CO_2_ or G×N interactions are noted in each panel.

Both lineages responded with increased growth to elevated CO_2_ (CO_2_, F_1,51_ = 13.70, P<0.001), N addition (N, F_1,51_ = 44.73, P<0.001), and CO_2_+N (CO_2_×N,F_1,51_ = 4.17, P = 0.047), but the introduced lineage had a greater overall growth response (G, F_1,51_ = 8.74, P = 0.005) and greater plastically to N (G×N, F_1,51_ = 6.51, P = 0 = 0.014) (Figure 1A, 2A). The vigorous growth response of the introduced lineage can be attributed to its inherently greater RGR (G,F_1,51_ = 29.73, P<0.001), which was enhanced by both CO_2_ (F_1,51_ = 11.34, P = 0.002) and N (F_1,51_ = 25.14, P<0.001) (Figure 1B). In turn, inherently greater productivity under current and predicted global change conditions may be attributed to the greater plant nitrogen productivity (NP) of the introduced type (G, F_1,51_ = 24.73, P<0.001), and by the fact that the NP of the introduced genotype increased under low N availability (G×N, F_1,51_ = 4.87, P = 0.032) (Figure 1C), whereas the NP of the native type was nearly static. Leaf C∶N indicated that the native type was more limited by N availability under all scenarios (G×N, F_1,51_ = 3.89, P = 0.055; G×CO_2_, F_1,51_ = 3.30, P = 0.076) (Figure 2M). Proportionally, biomass in the introduced was stimulated by 47% with CO_2_, 136% with N, and 319% with the combined CO_2_+N treatment. Due to a low growth under our control treatment (ambient CO_2_, low N) in the native genotype, biomass was stimulated 98% by CO_2_, 254% by N, and 510% by the combined treatment of CO_2_+N. Even though proportional stimulations were greater in the native, the introduced produced anywhere from 2.5 to 3.6 times more biomass within any given treatment.

Both CO_2_ and N stimulated all measured plant traits with two exceptions: SMF, RhMF, and R∶S changed only with N treatment, and SLA changed only with CO_2_ treatment (Table 1)(Figures 2A–O). CO_2_+N interactively stimulated total biomass (CO_2_×N, F_1,51_ = 4.15, P = 0.048) (Figure 2A), aboveground biomass (CO_2_×N, F_1,51_ = 5.39, P = 0.025)(Figure 2D), increased plant SMF (CO_2_×N, F_1,51_ = 8.15, P = 0.007)(Figure 2C) and decreased plant R∶S ratio (CO_2_×N, F_1,51_ = 4.90, P = 0.032) (Figure 2J). The introduced lineage had a greater response to all response variables (p<0.001) in both proportional and absolute terms (Table 1, Figures 1 and 2) except for SMF and LMF (Figures 2C and 2G).

**Table 1:** Summary of p-value results from factorial analysis of variance (ANOVA).

Biomass Stimulation	Nitrogen Productivity	AG Biomass	BG Biomass	Density	Root Mass	Rhizome Mass	Leaf Canopy	LAR	SLA	SMF	LMF	RMF	RhMF	R∶S	Stem Diameter
**<0.001**	**<0.001**	**<0.001**	**<0.001**	**<0.001**	**<0.001**	**<0.001**	**<0.001**	**<0.001**	**<0.001**	**<0.001**	**0.004**	**<0.001**	**<0.001**	**<0.001**	**<0.001**
**<0.001**	**<0.001**	**<0.001**	**<0.001**	**0.017**	**<0.001**	**<0.001**	**0.033**	**<0.001**	**<0.001**	0.115	**<0.001**	**0.045**	0.469	0.092	**<0.001**
**<0.001**	0.142	**<0.001**	**<0.001**	**<0.001**	**0.001**	**<0.001**	**<0.001**	**<0.001**	0.876	**<0.001**	**<0.001**	**<0.001**	**0.0366**	**<0.001**	**<0.001**
**0.047**	0.508	**0.025**	0.177	0.272	0.365	0.114	0.102	0.934	0.263	**0.007**	0.258	0.631	0.083	**0.032**	0.266
0.134	0.509	0.260	**0.039**	0.100	**0.023**	0.059	0.424	0.179	**0.002**	0.670	0.590	**0.014**	0.125	0.444	0.053
**0.014**	**0.032**	**0.009**	**0.046**	**0.042**	0.299	**0.016**	**0.002**	0.141	0.374	0.424	0.166	**0.026**	0.922	0.344	0.120

P values less than or equal to 0.05 are bold.

Most importantly, the introduced lineage exhibited greater fitness as evidenced by the greater mean trait value in all the response variables as interpreted by a significant G×CO_2_ or G×N interaction (Figure 2A–O, Table 1). As a whole, the introduced was more plastic to N than the native by producing more total biomass (G×N, F_1,51_ = 6.57, P = 0.014) (Figure 2). N fertilization also induced a more plastic stimulation aboveground (G×N, F_1,51_ = 7.58, P = 0.009) (Figure 2D), which was achieved by a threefold increase in ramet density (G×N, F_1,51_ = 4.41, P = 0.042)(Figure 2B). Only the introduced lineage optimized leaf structure and plant photosynthetic canopy by decreasing SLA in response to elevated CO_2_ (G×CO_2_, F_1,51_ = 11.47, P = 0.002)(Figure 2N), increasing the leaf area with N (G×N, F_1,51_ = 11.26, P = 0.002)(Figure 2E) and growing taller (G, F_1,51_ = 5.53, P = 0.023)(Table 1). Although native *Phragmites* allocated proportionally more mass to leaves (LMF)(Figure 2F), due to a greater SLA, LAR was still greater in the introduced (G, F_1,51_ = 106.21, P<0.001)(Figure 2O) maximizing photosynthetic gain.

Belowground productivity of the introduced *Phragmites* genotype responded to the four resource treatments more strongly than the native genotype in both proportional (LMF, SMF, RMF, & RhMF) and absolute terms. For example, introduced *Phragmites* produced between 332% to 541% more belowground biomass than the native type across the four resource treatments (Figure 2g), increasing roots in response to CO_2_ or decreasing roots in response to N (G×N, F_1,51_ = 4.20, P = 0.0465). This was true whether root biomass was expressed as mass (G×CO_2_, F_1,51_ = 4.53, P = 0.040; G×N, F_1,51_ = 4.20, P = 0.047) (Figure 2h) or a fraction of total plant biomass (RMF) (G×CO_2_, F_1,51_ = 6.51, P = 0.014; G×N, F_1,51_ = 5.33, P = 0.026)(Figure 2I), demonstrating a breadth of plasticity with respect to both global change factors. In addition, the introduced lineage allocated more resources to clonal expansion and carbohydrate storage in rhizomes under both elevated N (G×N, F_1,51_ = 6.74, P = 0.0128) and elevated CO_2_ (G×CO_2_, F_1,51_ = 3.77, P = 0.0585) (Figure 2K & 2L). This overall greater investment belowground resulted in below∶aboveground ratios for the native lineage that were about half those of the introduced lineage under any given treatment (G, F_1,51_ = 68.23, P<0.001)(Figure 2J).

**Figure 3:**
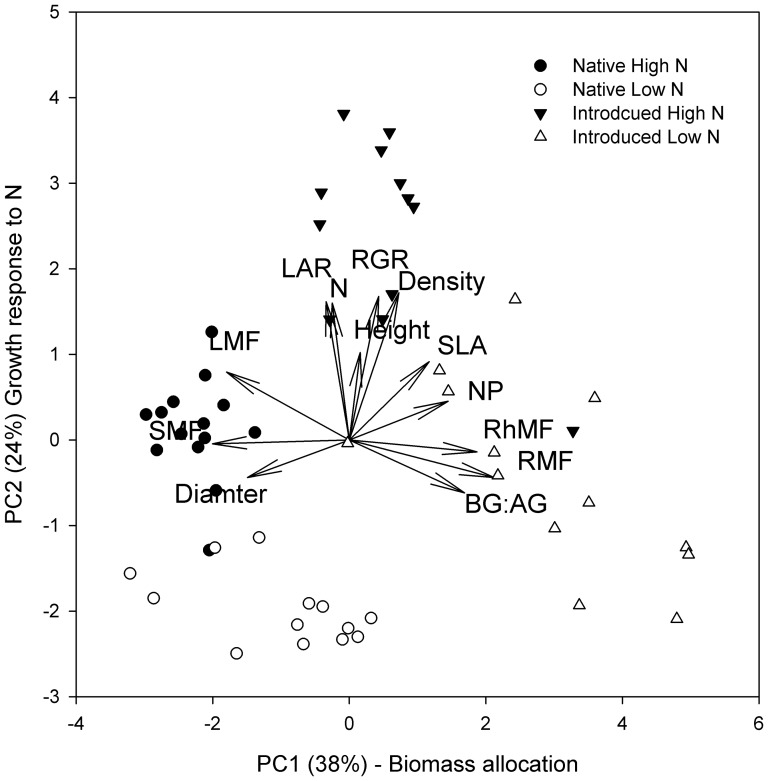
Principal component analysis of plant phenotype in response to CO_2_ and N treatments. Circles indicate native *Phragmites* and triangles indicate introduced *Phragmites*. Filled symbols are indicative of N-treated plants. Axis 1 is interpreted as a biomass allocation and Axis 2 is interpreted as growth response to N.

Our PCA indicated that plant lineages responded differently to global change, and lineages were aligned orthogonally to each other. The first two principal components accounted for 62% of the total variation in the data with eigenvalues of 4.95 and 3.13 for principal components 1 and 2, respectively (Figure 3, Table 1). Positive values on axis PC1 were related to a greater investment in belowground biomass allocation (R∶S, RMF, RhMF, introduced type), whereas negative values indicated an investment in slower growing, high investment tissue (SMF, stem diameter; native type) and explained 38% of the total variation. The second axis explained 24% of the total variation with PC2 representing the plant response to N, with positive values indicative of traits that were responsive to N and fast growth (LAR, ramet density, RGR, leaf area, height, and leaf N content; introduced lineage). Greater NP and SLA were traits unique to introduced *Phragmites*.

## Discussion

Our results are the first to demonstrate the potential importance of jack-and-master phenotypic plasticity to field-relevant global change variables, and provide insights to the traits that foster plant invasions. The introduced genotype had greater trait expression (e.g. greater biomass) across all treatments, showing that it was more fit than the native genotype across a wide range of resource availability, the definition of a jack-and-master. The greater fitness of the introduced genotype under the comparative stress of resource limitation (ambient CO_2_, low N) can be interpreted as a non-plastic response to selection in its native environment. The introduced lineage also showed greater change in the expression of several important traits with increasing resource availability (elevated CO_2_ and/or N), which is a plastic fitness response. This particular combination of responses – greater fitness regardless of resource availability, but greater plasticity under some resource environments and not others – is defined as a jack-and-master strategy. This was particularly evident for biomass production under scenarios of elevated CO_2_ and N that were more than 2-fold greater in the introduced lineage than the native lineage under all tested environmental conditions. Our results are consistent with two previous studies that demonstrated a greater growth response from the introduced lineage when exposed to either N [28] or salinity [22]. This suggests, but does not demonstrate, that elevated CO_2_ and N eutrophication will favor the introduced lineage in areas where it competes with the native lineage. We also speculate that the robust response of the introduced lineage to elevated CO_2_ may help it compete against native C_4_ grasses that respond weakly to elevated CO_2_ [24].

The invasion success of *Phragmites* under current environmental conditions has been attributed to its inherently high relative growth rates, biomass, and stature under contemporary environmental conditions [21], [22]. We propose that global change in the form of elevated CO_2_ and ongoing N eutrophication will further promote invasion in North American wetlands. We acknowledge that greater growth potential alone cannot solely predict invasion success because traits like fecundity, resistance to herbivory, and tolerance of abiotic factors may influence both growth and expansion. However, in the conspecific comparison used here, introduced *Phragmites* seeds have over a three times greater germination rate [27], and recent research suggests that *Phragmites* spreads primarily sexually by seed [34]. In North America, herbivores have been found to prefer native *Phragmites* [35], [36], giving the introduced conspecific a competitive advantage. Finally, ecophysiological trials have indicated that introduced *Phragmites* is more salt tolerant [22] and more efficient at oxygenating its rhizosphere [37] than the native, providing access to a greater range of wetland environments and potentially greater drought tolerance. Because these factors provide advantages to the introduced conspecific in addition to elevated CO_2_ and N, we suggest that the greater growth we observed will likely increase the competitive ability of the introduced genotype by facilitating colonization and rapid spread through clonal integration.

The ability to change nutrient-use strategies among our four simulated environments is a mechanism that may explain the robust growth response of the introduced lineage. Based on the nitrogen economy of the two lineages [3], [21], we expected the native lineage to perform strongly in the low N treatment, and perhaps outperform the introduced lineage. However, only the introduced lineage exhibited a statistically plastic nitrogen productivity (NP) response to the N treatments, becoming especially N efficient in the low N treatment. This suggests it can optimize N use under a variety of scenarios, consistent with our designation of the plant as a jack-and-master. This observation is relevant to the well established fact that N availability constrains plant growth responses to elevated CO_2_ [25]. Acknowledging the caveat that our experiment was of short duration, we interpret the increased NP under both low N conditions and elevated CO_2_ to suggest that N limitation in a field setting is unlikely to eliminate the elevated CO_2_ stimulation of *Phragmites* growth reported here in a greenhouse setting.

Plasticity in specific leaf area (SLA) to elevated CO_2_ may also explain differences in plant responses across our global change treatments. Typically, plants grown at elevated CO_2_ have reduced SLA and improved water use efficiency [38]; however, only the introduced lineage showed reduced SLA, apparently optimizing leaf structure for growth under elevated CO_2._. We previously suggested that the greater SLA of the introduced lineage is one factor responsible for its greater relative growth rate (RGR) and invasive success [21]. We now suggest that the introduced lineage's ability to optimize SLA under elevated CO_2_ also translates into a greater leaf area ratio (LAR) and plant RGR [32], which may further facilitate its competitive ability and invasion with predicted global change.

Our data also suggest that inherently fast growth (high RGR) may contribute to a large CO_2_ response in other invasive species. In the present study, differences between lineages with respect to RGR (Figure 1) and phenotypic plasticity corresponded to their respective responses to elevated CO_2_. In particular, the inherently higher RGR of the introduced lineage can be attributed to its greater capacity and plasticity in several plant traits that contribute to rapid growth above and belowground, including SLA, NP, LAR, and root mass fraction (RMF). In contrast, the native lineage allocated more biomass to stem mass (SMF), indicative of plants with lower growth rates [32]. Although this is far too small a sample size on which to draw generalizations, our observations suggest a line of inquiry into the relationship between easily quantified metrics of plant growth from which predictions of invasive success under global change conditions could be made.

The ability to plastically change biomass allocation to favor RMF under both elevated CO_2_ and N is an unseen plastic trait. Holdredge *et al.* [39] reported that plastic root architecture and the presence of mycorrhizal symbionts allowed the native type to compete well under nutrient limited conditions. In our study, we did not assess phenotypic differences in either root length or mycorrhizal status, but the native type produced less root mass (in both proportional and absolute terms) and had a higher foliar C∶N ratio than the introduced lineage. These data suggest that the more efficient root system of the native lineage [23] could not provide enough N to satisfy plant N demand and growth in comparison to the introduced lineage.

The responses of introduced and native *Phragmites* lineages to variation in resource availability support the hypothesis that invading species are more plastic in adaptive traits than native species [9]. In particular, the introduced lineage was more plastic in nearly all traits measured (as indicated by a significant G×E interaction) except for R∶S, LAR, RhMF, LMF, and SMF. For these traits, the introduced lineage had significantly greater R∶S, LAR, and RhMF, and significantly lower LMF and SMF than the native lineage. Although elevated CO_2_ caused a shift to belowground structures in both lineages – a common response in C_3_ plants [40] – proportionally the introduced lineage was more plastic by optimizing RMF in response to both CO_2_ (by increasing) and N (by decreasing). Furthermore, the introduced invested proportionally two times more biomass belowground. In clonal species, such as *Phragmites*, resource sharing through belowground structures facilitates plant invasion through increased carbohydrate storage and clonal expansion [41]. We suggest that introduced *Phragmites*, and perhaps other invasive species that are plastic in terms of biomass allocation, will likely be an increasingly successful invader as atmospheric CO_2_ concentrations continue to rise, although the size of the elevated CO_2_ effect may interact with N availability.

N availability, more so than CO_2_ fertilization, limited the growth of both *Phragmites* lineages under the edaphic and environmental conditions of our experiment. However, N availability was more limiting to the native type (higher C∶N, lower NP), the one scenario that we predicted would favor the native type [3], [21]. We added N at a rate similar to current levels of N pollution in coastal marshes of the eastern United States [30] and observed a more plastic stimulation of biomass production in the introduced lineage. These data support the hypothesis that N has contributed to increases in introduced *Phragmites* distribution in anthropogenically modified environments [42], [43]. The lesser growth response of native *Phragmites* to N, also observed by Saltonstall and Stevenson [28], may be explained by greater investment in slower-growing individual units (low density, high SMF), indicative of plants adapted to low nutrient environments, and to a relative lack of plasticity in the plant NP response.

How invasive species respond to multiple interacting global change factors is largely unknown at the community and ecosystem level. Our PCA analysis suggests that the introduced lineage has the potential for both greater aboveground and belowground growth in response to changes in the balance of C (as CO_2_) and N availability. In contrast, experiments on native upland and wetland species have suggested that elevated CO_2_ favors certain species, while added N favors different species [26]. As a result, adding N can actually diminish the elevated CO_2_ response at an ecosystem-level [26]. Our results support the proposal by Langley and Megonigal [26] that elevated CO_2_ and N will increase the invasion success of *Phragmites* because it may respond more positively to both factors simultaneously compared to competing species. This “jack-and-master” plasticity in response to global change variables may provide an advantage to introduced *Phragmites* in competition with native species that are subject to resource tradeoffs in which a strong response to one global change factor precludes a strong response to another.

There are few instances of “jack-and-master” invaders in the literature [7], [9] and such invaders may reflect a relatively rare strategy in nature. Detecting a jack-and-master genotype requires information on both phenotypic plasticity (i.e. changes in trait expression) and plant performance (i.e. absolute expression of a trait such as biomass). In our controlled experiment, the introduced lineage outperformed the native lineage under all environmental conditions, and it also exhibited greater plasticity with respect to plant NP, SLA, and RMF. We suggest that the invasive success of the introduced lineage is due to both inherently favorable non-plastic traits such as RGR, and trait plasticity in SLA and NP that allowed the introduced lineage to maximize RGR under both stressful and favorable conditions. The combination of these factors may help explain its expansion into historically unoccupied habitats throughout North America.

While we have focused on the greater fitness response of the introduced genotype in terms of invasiveness, it must also be noted that the native genotype responded (1) positively to both global change factors and (2) proportionally more from the control treatment than the introduced. This suggests that the native type may also become more vigorous with predicted global change. Regardless, the greater proportional response in biomass (due to the poor performance under the control scenario) of the native is overshadowed by the greater fitness response of the introduced (2 to 3 time more biomass) suggesting that proportional responses may not be as important as absolute responses in terms of growth and invasiveness. More recently, several studies have questioned the role of phenotypic plasticity in plant invasions, and it has been suggested that inherently greater mean trait values may trump phenotypic plasticity [44], [45]. The higher mean trait values we reported combined with a plastic response with increasing resource availability are consistent with our observed jack and master strategy. Godoy *et al* [44] suggested that new plasticity indices be developed that combine plasticity with mean trait values to evaluate the combined role of plasticity and mean trait values for invasive plant success.

Because there was one population per genetic lineage, the results of our study do not take account of variation among native and introduced populations in their response to elevated CO_2_ and nitrogen. While clone specific differences are likely within each lineage, and examples exist in *Phragmites* [46]–[48], it is unclear if these clone specific differences can be attributed to either genotype or genetic lineages since these studies were prior to the knowledge of distinct lineages [17]. However, our observed trait differences between the native and introduced populations in our control treatment were consistent with similar comparisons reported in the literature [21], [22], [28]. Thus, we assert that our results and interpretations may be cautiously generalized to other North American and European lineages. Our experiment addressed short-term (1 growing season) responses to global change factors, and therefore does not account for relatively long-term responses such as photosynthetic acclimation to elevated CO_2_ [49] and progressive nitrogen limitation [25]. A longer greenhouse experiment was not possible because of the well-documented effects of pot volume on CO_2_ responses [50], which could have confounded our results and interpretations, as was likely the case in a previous study that reported no effect of elevated CO_2_ on *P. communis* [51]. Although we can anticipate that long-term responses may be less than short-term responses [52], it is clear from many years of elevated CO_2_ experimentation that short-term responses adequately predict the direction of the long-term responses.

### Consequences of global change driven invasion in Atlantic coast wetlands

Over the next century, CO_2_ concentrations are expected to exceed 700 ppm (IPCC 2007), which will likely stimulate the growth of this invasive lineage, surpassing the invasion success that is observed today. Our data suggest that the combination of elevated CO_2_ coupled with N eutrophication has the potential to increase *Phragmites* productivity beyond current levels. While it has been suggested that elevated CO_2_ may mitigate the loss of species diversity attributed to N pollution [53], we do not believe this prediction will apply to ecosystems subject to invasion by species like *Phragmites*. Introduced *Phragmites* has a higher affinity for N and outcompetes C_4_ grasses for N [23], it can plastically change NP, and it is also a better competitor for light than adjacent native species [42]. Increasing CO_2_ concentrations will likely increase plant water use efficiency [54], potentially allowing it to invade into other presently unoccupied niches of greater salinity or flooding.

Although invasion dramatically changes plant communities, global change-driven invasion of *Phragmites* may also have unanticipated benefits. For example, the 330–541% greater belowground growth of introduced *Phragmites* will most likely lead to greater soil carbon sequestration, resulting in the ecosystem becoming a greater sink for C. In coastal wetlands under the threat of accelerating relative sea level rise, which may drown many coastal marshes world-wide within the next century [55], elevated CO_2_ can stimulate soil elevation gain [6], counteracting the effects of relative sea level rise. Current rates of soil elevation gain in *Phragmites*-dominated marshes can be greater than the native-dominated marshes it replaces [56], and our data suggest that elevated CO_2_ and N will likely enhance elevation gain further by raising surface elevation through direct organic matter addition. As such, we suggest that marshes dominated by invasive *Phragmites* may have an improved chance of increasing elevation rapidly enough to keep pace with accelerated relative sea level rise.

Our data suggests that, even in a high CO_2_ environment, it may be possible to limit future *Phragmites* invasions if wetland ecosystems can be restored to their pre-industrial oligotrophic state. These findings are important to land managers currently dealing with rampant *Phragmites* invasion. Assuming global changes in CO_2_ are inevitable given the current infrastructure of our global energy supply, the only tractable and manageable variable impacting future *Phragmites* invasion is N pollution. Limiting anthropogenic N pollution, even in a high CO_2_ world, may limit future *Phragmites* invasion due to N limitation, and potentially prevent catastrophic losses in species richness and ecosystem services. However, if management practices to control N loading are not set in place, it is likely that introduced *Phragmites* invasion will intensify in the future and surpass the levels of invasion observed today.
